# Gut Microbiota Dysbiosis in Acute Ischemic Stroke Associated With 3-Month Unfavorable Outcome

**DOI:** 10.3389/fneur.2021.799222

**Published:** 2022-01-28

**Authors:** Huanhuan Sun, Mengmeng Gu, Zhongyuan Li, Xiangliang Chen, Junshan Zhou

**Affiliations:** Department of Neurology, Nanjing First Hospital, Nanjing Medical University, Nanjing, China

**Keywords:** gut microbiota, 16S rRNA, dysbiosis, ischemic stroke, functional outcome

## Abstract

**Background:**

Alterations in the gut microbiota after ischemic stroke have been demonstrated, whereas the effect on stroke outcome remains to be established.

**Methods:**

A total of 132 consecutive patients with acute ischemic stroke were prospectively enrolled. Their gut microbiomes within 24 h of admission were profiled using 16S ribosomal RNA (rRNA) gene (V3–V4 region) sequencing. Microbiota comparisons were made between groups with good outcome (*n* = 105) and poor outcome (*n* = 27) based on 3-month modified Rankin Scale scores of 0–2 and 3–6. Propensity score-matching (PSM) analysis was conducted to assess the robustness of our findings. The functional potential was predicted using the Phylogenetic Investigation of Communities by Reconstruction of Unobserved States (PICRUSt).

**Results:**

Patients in the poor outcome group were characterized by a significant reduction in the alpha diversity (Shannon index, *p* = 0.025; Simpson index, *p* = 0.010), an increase in the pathogenic bacteria (e.g., *Enterococcaceae* and *Enterococcus*), and a decrease in the short-chain fatty acids (SCFAs)-producing bacteria (e.g., *Bacteroidaceae, Ruminococcaceae*, and *Faecalibacterium*) to those with good outcome group (all *p* < 0.05). Similar results of microbial composition were obtained after PSM. The PICRUSt revealed that the pathway for membrane transport was relatively dominant in patients with poor outcome (*p* < 0.05).

**Conclusion:**

This study demonstrated that stroke patients with 3-month poor outcome had baseline gut microbiota dysbiosis featured by increased pathogenic bacteria and decreased SCFAs-producing bacteria.

## Introduction

Stroke is one of the leading causes of disability and death, especially in the population aged 50 years and older (1). For acute ischemic stroke, about one-third of patients die or become disabled within the first 3 months (2). Identifying prognostic factors in the acute phase of stroke is important and might provide a novel therapeutic target to improve the stroke outcome.

Evidence is emerging that the gut microbiota is intimately involved in the pathology of a wide range of neurological disorders, including acute ischemic stroke (3). This is supported by the observations that gut microbiota correlates closely with stroke risk factors, such as age, obesity, hypertension, diabetes, dyslipidemia, and atrial fibrillation (4–8). Moreover, recent studies described a consistent pattern of poststroke gut microbiota dysbiosis, which is characterized by an increase in opportunistic pathogens (e.g., *Enterobacteriaceae*) and a decrease in commensal bacteria (e.g., *Fecalibacterium*), yielding a pro-inflammatory effect (9–14). Besides, gut microbiota-derived metabolites such as trimethylamine-N-oxide (TMAO) and short-chain fatty acids (SCFAs) might contribute to stroke pathology by modulating inflammation and atherosclerosis (11, 15–18). The SCFAs could regulate the expression of brain-derived neurotrophic factor (BDNF) (16); they could also modulate the effects of brain-invading lymphocytes, leading to an altered level of proinflammatory interleukin-17 (IL-17) and neuroprotective IL-10 (15). Interestingly, previous studies showed that the alterations of gut microbiota after stroke are correlated with the prognosis of stroke (10–13). The enrichment of *Parabacteroides, Oscillospira, Enterobacteriaceae*, etc., and depletion of *Prevotella, Roseburia, Fecalibacterium*, etc., were positively correlated with early unfavorable outcomes at discharge in patients with stroke (12). Whereas, the specific groups of bacteria and potential mediators associated with 3-month functional outcome in patients with acute ischemic stroke have not been fully revealed.

Here, we conducted this 16S ribosomal RNA (rRNA) gene sequencing-based study to characterize the gut microbiota in the acute phase of ischemic stroke and to compare the bacteria profiles of patients with different functional outcomes [3-month modified Rankin Scale (mRS) 0–2 vs. 3–6]. We aimed to: (1) reveal the taxonomic dysbiosis of gut microbiota related to unfavorable stroke outcome; (2) infer possible differences of functional composition associated with stroke outcome; (3) explore whether inflammatory cytokines (IL-17 and IL-10), BDNF, and TMAO are associated with gut microbiota and stroke outcome. An additional propensity score-matching (PSM) analysis was performed to correct for sample selection bias.

## Materials and Methods

### Participants and Sample Collection

The participants were consecutively recruited from May 2018 to June 2019 in the Department of Neurology, Nanjing First Hospital (a teaching hospital affiliated with Nanjing Medical University). The inclusion criteria have been described in detail in our previous work (19). In brief, we enrolled patients aged ≥ 50 years with acute ischemic stroke due to anterior cerebral infarction. The exclusion criteria were: radiological evidence of intracerebral hemorrhage; chronic inflammatory or immune diseases (e.g., rheumatoid arthritis, lupus erythematosus, inflammatory bowel disease, or chronic hepatitis); severe comorbidities or an unstable medical condition (e.g., congestive heart failure, respiratory failure, renal failure, or severe liver dysfunction); and consumption of probiotics, antibiotics, or immunosuppressants within 1 month before admission. This study was approved by the Ethics Committee of Nanjing First Hospital with the approval number 20180713-05 and all the participants (or their legal guardians) signed a written informed consent.

Demographic information and medical histories were collected shortly after admission through a face-to-face interview. The baseline National Institutes of Health Stroke Scale (NIHSS) and the Diffusion-Weighted Imaging-Alberta Stroke Program Early CT score (DWI-ASPECTs) were assessed by two neurologists (HS and ZL).

Fecal and fasting blood samples were taken within 24 h after admission. The fecal samples were collected using sterile fecal containers with a spoon and each sample weighed ~2 g. After collection, fresh fecal and serum samples were immediately (within 1 h) stored at −80°C until analysis. The fasting blood samples were collected after overnight fasting and measured at the hospital central laboratory for total cholesterol (TC), triglyceride (TG), high-density lipoprotein cholesterol (HDL), low-density lipoprotein cholesterol (LDL), and fasting blood glucose (FBG).

Functional outcomes were assessed by two neurologists (HS and ZL) through telephone interviews using the mRS score at 3 months after stroke. Three patients were lost to follow-up and were excluded. According to the 3-month mRS scores, participants were divided into the two groups: the good functional outcome group (mRS 0–2) and the poor functional outcome group (mRS 3–6).

### Fecal DNA Extraction

Fecal genomic DNA was extracted from the fecal samples with the QIAamp® DNA Stool Mini Kit (Qiagen, Hilden, Germany), according to the protocol of the manufacturer. The concentration and purity were detected through the Nanodrop, and the integrity was detected through regular 0.8% agarose gel electrophoresis.

### Polymerase Chain Reaction Amplification and Illumina Sequencing

The V3–V4 variable regions of the bacterial 16S rRNA gene were amplified by PCR using the forward (5′-CCTACGGGNGGCWGCAG-3′) and reverse (5′-GACTACHVGGGTATCTAATCC-3′) primers. The primary PCR products were checked by agarose gel electrophoresis and pooled to be used as the template for index PCR. The indexed PCR was performed by using index primers for adding the Illumina index to the library. After indexed PCR, the amplification products were checked and purified to be indexed in the 16S V3–V4 library whose quality was assessed on the Qubit@2.0 Fluorometer (Thermo Fisher Scientific, Waltham, Massachusetts, USA) and Agilent Bioanalyzer 2100 systems. Finally, the pooled library was sequenced on an Illumina MiSeq 250 Sequencer for generating 2 × 250 bp paired-end reads.

### Quantification of Serum TMAO, IL-17, IL-10, and BDNF

The concentration of serum TMAO level was determined using an enzyme-linked immunosorbent assay kit (MyBioSource Incorporation, San Diego, California, USA). The serum IL-17, IL-10, and BDNF levels were determined using enzyme-linked immunosorbent assay kits (R&D Systems Incorporation, Minneapolis, Minnesota, USA).

### Bioinformatics and Statistical Analysis

The raw reads were quality filtered and merged, and the remaining unique reads were chimera checked compared with the gold.fa database (http://drive5.com/uchime/gold.fa). These reads were clustered into operational taxonomic units (OTUs) by UPARSE with a cutoff of 97% similarity. All OTUs were classified based on Ribosomal Database Project by Mothur. Alpha diversity was analyzed by Mothur to describe the abundance and diversity of gut microbiota, including Observed species, Chao1, Abundance-based Coverage Estimator (ACE), Shannon, Simpson, and Coverage index. Beta diversity was utilized to further analyze the between-individual diversity of gut microbiota, using Principal Coordinates Analysis (PCoA) performed by R Project (Vegan package, version 3.3.1). Variation between groups was assessed by permutational multivariate ANOVA (PERMANOVA). Linear discriminant analysis (LDA) coupled with effect size (LEfSe) was conducted to determine the significantly differential taxa between the good outcome group and the poor outcome group. The absolute value of logarithmic LDA score > 2 and *p* < 0.05 were considered statistically significant. The Spearman correlation analysis was used to detect the associations between biochemical parameters and the relative abundance of microbiota. Functional metabolic profiles of bacterial communities were predicted using the Phylogenetic Investigation of Communities by Reconstruction of Unobserved States (PICRUSt).

Statistical analyses were performed with SPSS 22.0 for Windows (SPSS Inc., Chicago, Illinois, USA) and R version 3.3.1 (R Development Core Team, Vienna, Austria). Categorical variables were compared by the chi-squared test and continuous variables were compared by the Student's *t*-test or the Mann–Whitney *U*-test. The PSM analysis was performed with age, hypertension, and the baseline NIHSS as covariates. The corresponding propensity score of the grouping variable (mRS 0–2 or mRS 3–6) was calculated for each patient with a 1:1 nearest-neighbor matching algorithm (caliper width 0.2). Matched pairs of samples were obtained for analysis to adjust for sample selection bias. STROBE guidelines were the basis for reporting our results. The resulting *p*-values were adjusted using the Benjamini–Hochberg false discovery rate correction. A two-sided *p* < 0.05 was considered as statistically significant.

## Results

### Study Population

A total of 132 subjects (89 males and 43 females) aged 50–91 years (mean 64.5 years) were recruited for this study (Supplementary Figure S1). Patients in the mRS 3–6 (*n* = 27) group were older than those in the mRS 0–2 (*n* = 105) group (75.2 ± 10.9 vs. 65.9 ± 9.2, *p* = 0.001). Besides, patients in the poor outcome group had a higher incidence of hypertension and atrial fibrillation, higher baseline NIHSS scores, and lower DWI-ASPECT scores (*p* < 0.05). No group differences were detected in gender, diabetes mellitus, dyslipidemia, and coronary heart disease. Also, no group differences were observed in medication histories including the use of anticoagulants, antiplatelet, statins, antihypertensive drugs, and antidiabetic agents. Small vessel occlusion was more frequent in the mRS 0–2 group, while the mRS 3–6 group was more likely to have other etiologies (including cardioembolism, the stroke of other determined cause, and stroke of undetermined cause). After PSM, there were no significant differences in demographic and clinical data between the two groups (Table 1).

**Table 1:** Characteristics of the study participants.

	**Before PSM**			**After PSM**		
**Characteristics**	**mRS 0-2** **(***N*** = 105)**	**mRS 3-6** **(***N*** = 27)**	***P*-value**	**mRS 0-2** **(***N*** = 17)**	**mRS 3-6** **(***N*** = 17)**	***P*** **-value**
Age, y, mean ± SD	65.9 ± 9.2	75.2 ± 10.9	0.001	70.9 ± 10.6)	71.9 ± 11.3	0.489
Male, *n* (%)	74 (70.5)	15 (55.6)	0.140	11 (64.7)	13 (76.5)	0.708
Hypertension, *n* (%)	82 (78.1)	26 (96.3)	0.029	15 (88.2)	17 (100.0)	0.485
DM, *n* (%)	43 (41.0)	6 (22.2)	0.072	5 (29.4)	3 (17.6)	0.688
Dyslipidemia, *n* (%)	12 (11.4)	1 (3.7)	0.230	9 (52.9)	13 (76.5)	0.282
CHD, *n* (%)	9 (8.6)	4 (14.8)	0.332	3 (17.6)	0 (0.0)	0.227
AF, *n* (%)	19 (18.1)	12 (44.4)	0.004	6 (35.3)	5 (29.4)	1.000
Medication history						
Anticoagulants (*N* = 131)	4 (3.8)	1 (3.8)	0.993	0 (0)	0 (0)	-
Antiplatelet (*N* = 125)	14 (13.9)	3 (12.5)	0.861	3 (18.8)	1 (7.1)	0.602
Statins (*N =* 131)	9 (8.6)	1 (3.8)	0.417	2 (11.8)	1 (6.2)	1.000
Anti-hypertensive drugs	56 (53.3)	15 (55.6)	0.836	11 (64.7)	10 (58.8)	1.000
Antidiabetic agents	24 (22.9)	3 (11.1)	0.177	4 (23.5)	1 (5.9)	0.335
TOAST, *n* (%)			0.016			0.747
LAA	42 (40.0)	11 (40.7)		7 (41.2)	9 (52.9)	
SVO	41 (39.0)	4 (14.8)		3 (17.6)	3 (17.6)	
Others	22 (21.0)	12 (44.4)		7 (41.2)	5 (29.4)	
TC (*N =* 131), median (IQR), mmol/L	4.3 (3.6-5.0)	4.4 (3.9–4.6)	0.744	3.6 (3.1–4.5)	4.4 (3.9–4.6)	0.290
TG (*N =* 131), median (IQR), mmol/L	1.4 (1.0–1.7)	0.9 (0.8–1.2)	0.003	1.3 (0.7–1.9)	0.9 (0.8–1.3)	0.085
HDL (*N =* 131), median (IQR), mmol/L	1.1 (0.9–1.2)	1.2 (1.1–1.4)	0.120	1.0 (0.9–1.2)	1.2 (1.1–1.4)	0.205
LDL (*N =* 131), median (IQR), mmol/L	2.4 (1.9–3.1)	2.7 (2.1–3.1)	0.814	2.4 (2.0–3.0)	2.8 (2.2–3.1)	0.413
FBG (*N =* 128), median (IQR), mmol/L	5.0 (4.4–6.1)	6.0 (5.3–7.6)	0.039	5.6 (4.7–6.4)	6.1 (5.8–8.0)	0.224
NIHSS–baseline, median (IQR)	3.0 (1.5–4.0)	13.0 (9.0–17.0)	0.001	14.0 (4.5–17.5)	12.0 (10.0–16.0)	0.812
DWI–ASPECTs (*N =* 131), median (IQR)	8.0 (7.0–9.0)	6.0 (3.5–8.3)	0.001	8.0 (4.5–8.5)	6.5 (4.5–9.0)	0.817
TMAO (*N =* 131), median (IQR), μmol/L	2.8 (1.5–4.1)	2.2 (1.4–4.0)	0.513	1.8 (0.7–2.7)	2.2 (1.2–3.7)	0.496
IL−10, median (IQR), μg/L	48.0 (30.9–64.3)	54.6 (37.0–67.2)	0.333	32.5 (23.4–45.7)	60.0 (37.0–68.4)	0.079
IL−17, median (IQR), ng/L	78.5 (65.5–94.0)	74.1 (63.7–95.6)	0.571	75.2 (60.6–95.9)	63.7 (57.6–103.2)	0.708
BDNF, median (IQR), μg/L	2.8 (2.0–3.5)	3.2 (2.2–3.7)	0.196	2.6 (1.9–3.2)	3.5 (2.7–4.1)	0.474

*Data were presented as median (interquartile range, IQR), mean ± SD, or number (%)*.

*PSM, propensity score–matching analysis; mRS, modified Rankin Scale; SD, standard deviation; DM, diabetes mellitus; CHD, coronary heart disease; AF, atrial fibrillation; TOAST, Trial of Org 10172 in Acute Stroke Treatment; LAA, large artery atherosclerosis; SAO, small artery occlusion; TC, total cholesterol; TG, triglyceride; HDL, high-density lipoprotein cholesterol; LDL, low-density lipoprotein cholesterol; FBG, fasting blood glucose; CRP, C-reaction protein; NIHSS, National Institute of Health Stroke Scale; DWI-ASPECTs, DWI-Alberta Stroke Program Early CT score; TMAO, trimethylamine N-Oxide; IL, interleukin; BDNF, brain-derived neurotropic factor*.

### Comparison of Gut Microbiota Diversity

**Figure 1:**
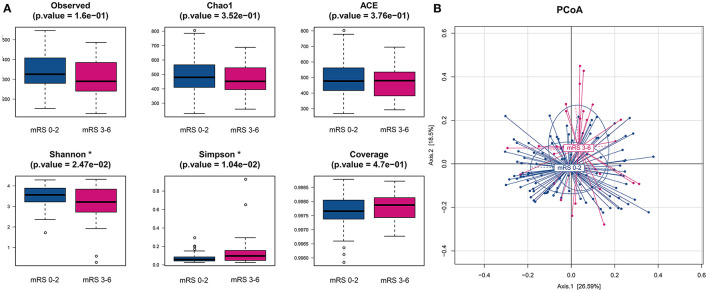
Comparison of alpha- and beta-diversities of gut microbiota between the two groups. **(A)** Six indices were used to represent the alpha-diversity: observed species, Chao1, Abundance-based Coverage Estimator (ACE), Shannon, Simpson and Coverage. **(B)** Principal coordinates analysis (PCoA) of the weighted uniFrac distances for the mRS 0–2 and mRS 3–6 subjects. The two components explain 26.6 and 18.5% of the variance, respectively. A significant separation was found between the two groups (*p* < 0.001, permutation test with pseudo-F ratios). Blue, mRS 0–2 subjects (*n* = 105); red, mRS 3–6 subjects (*n* = 27). mRS, modified Rankin Scale.

We obtained a total of 22,167,731 high-quality reads, which were clustered into 2,596 OTUs at 97% identity. The alpha-diversity analyses, including species richness (represented by observed species, Chao1, ACE) and evenness (represented by Shannon index and Simpson index) of the microbial community, showed that the mRS 3–6 group had less diversity than the mRS 0–2 group. There were statistically significant differences in the Shannon and Simpson indices between the two groups (mRS 0–2 vs. mRS 3–6: median Shannon index, 3.56 vs. 3.21, Wilcoxon *p* = 0.025; median Simpson index, 0.06 vs. 0.10, Wilcoxon *p* = 0.010). The observed species and Chao1 were lower in the mRS 3–6 group, but the differences between groups did not reach statistical significance (observed species, *p* = 0.160; Chao1, *p* = 0.352) (Figure 1A).

To compare the differences in microbial structure, PCoA was used (based on weighted-unifrac distance) for beta diversity analysis. The Adonis test was used for statistical significance test and revealed an obvious dissimilarity between the mRS 0–2 and mRS 3–6 groups (Adonis test, *p* < 0.001, Figure 1B).

### Taxonomic Differences of Gut Microbiota

**Figure 2:**
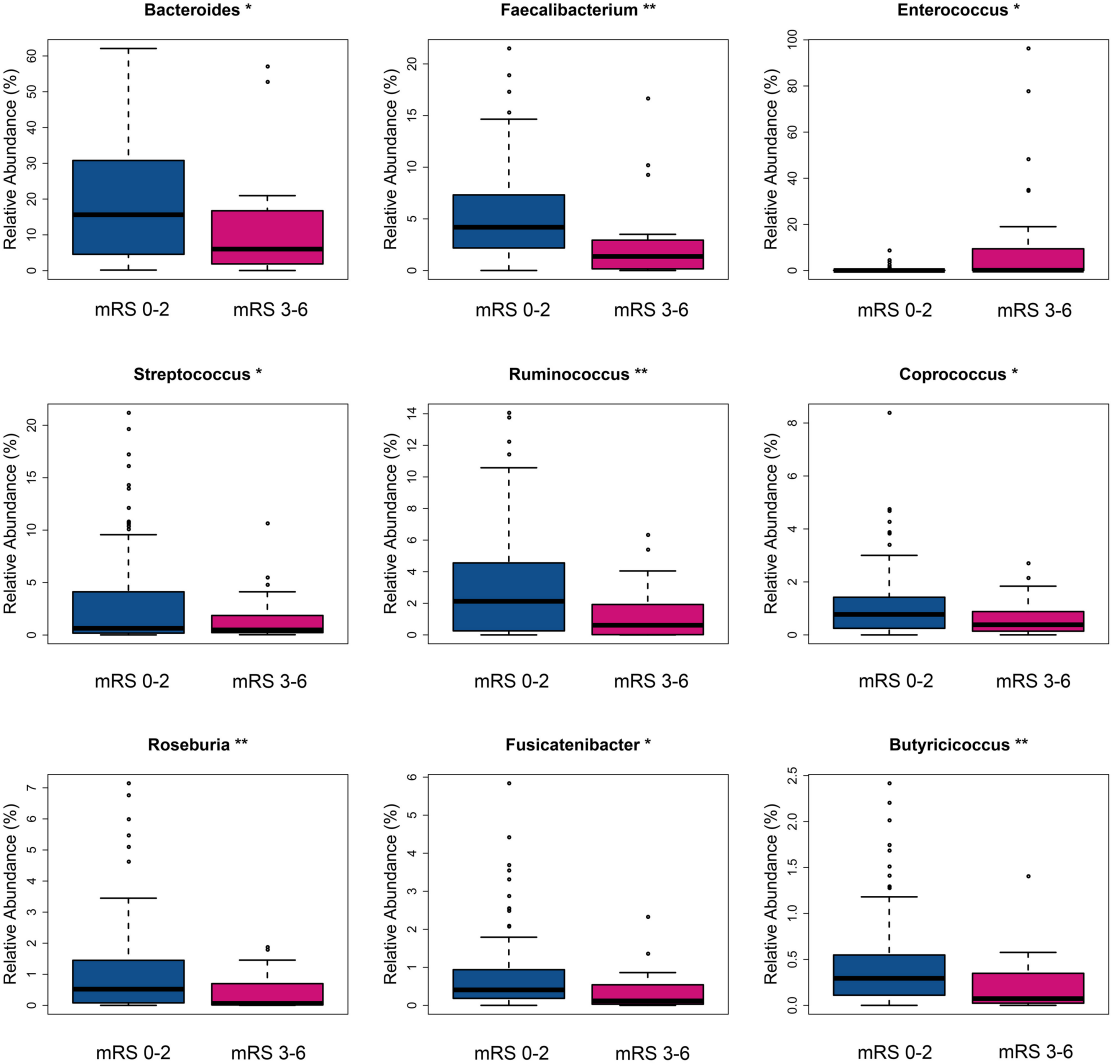
Taxa with significantly different relative abundance between two groups at the genus level. *p < 0.05, **p < 0.01. mRS, modified Rankin Scale.

**Figure 3:**
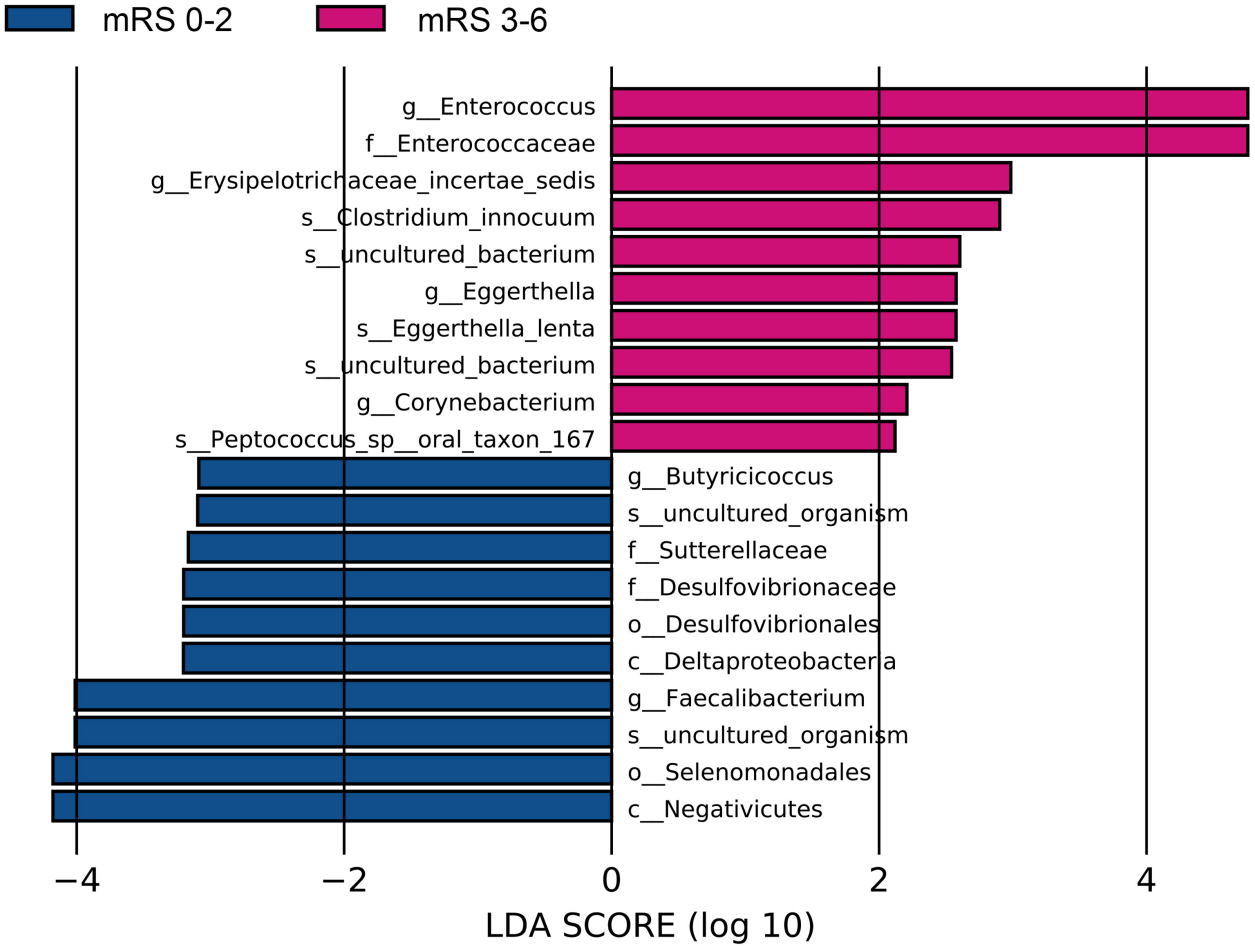
Significantly discriminant taxa between the good outcome and poor outcome patients determined using linear discriminate analysis size effect (LEfSe). LEfSe, linear discriminate analysis size effect; LDA, linear discriminate analysis; mRS, modified Rankin Scale.

The fecal bacteria communities detected in both groups were dominated by 4 main phyla (Supplementary Figure S2A), including *Firmicutes, Bacteroidetes, Proteobacteria*, and *Actinobacteria*. The dominant families that account for more than 80% abundance of the total bacteria were *Lachnospiraceae, Bacteroidaceae, Ruminococcaceae, Enterobacteriaceae*, and *Bifidobacteriaceae* (Supplementary Figure S2B). The relative abundance of bacteria taxa varied between the two groups. At the phylum level, the gut microbiota in the mRS 3–6 group showed a decrease of beneficial bacteria *Bacteroidetes* (mean relative abundance, mRS 3–6 group 19.0 vs. mRS 0–2 group 29.8%; *p* = 0.012, Supplementary Figure S3A). At the family level, beneficial or common core bacteria *Bacteroidaceae, Ruminococcaceae, Veillonellaceae*, and *Streptococcaceae* were depleted, while pathogenic bacterium *Enterococcaceae* was enriched in the mRS 3–6 group, though another opportunistic pathogen *Desulfovibrionaceae* was depleted (Supplementary Figure S3B). At the genus level, the abundance of six well-known SCFAs-producing genera, *Bacteroides, Faecalibacterium, Roseburia, Ruminococcus, Coprococcus*, and *Butyricicoccus*, as well as the other two genera, *Streptococcus* and *Fusicatenibacter* were lower in the mRS 3–6 group, while in contrast, the pathogenic bacterium *Enterococcus* was enriched in this group (Figure 2). Further analysis using LEfSe revealed higher abundances of pathogenic bacteria *Enterococcaceae, Enterococcus*, and *Eggerthella*, as well as a lower abundance of SCFAs bacteria *Faecalibacterium* and *Butyricicoccus* in the mRS 3–6 group (Figure 3). After PSM, the mRS 3–6 group still showed less abundant SCFAs-producing bacteria of *Ruminococcus* and *Faecalibacterium*, and more abundant pathogenic bacterium of *Enterococcus* (Supplementary Figures S4, S5).

### Gut Microbiota Dysbiosis in Association With Clinical Characteristics

**Figure 4:**
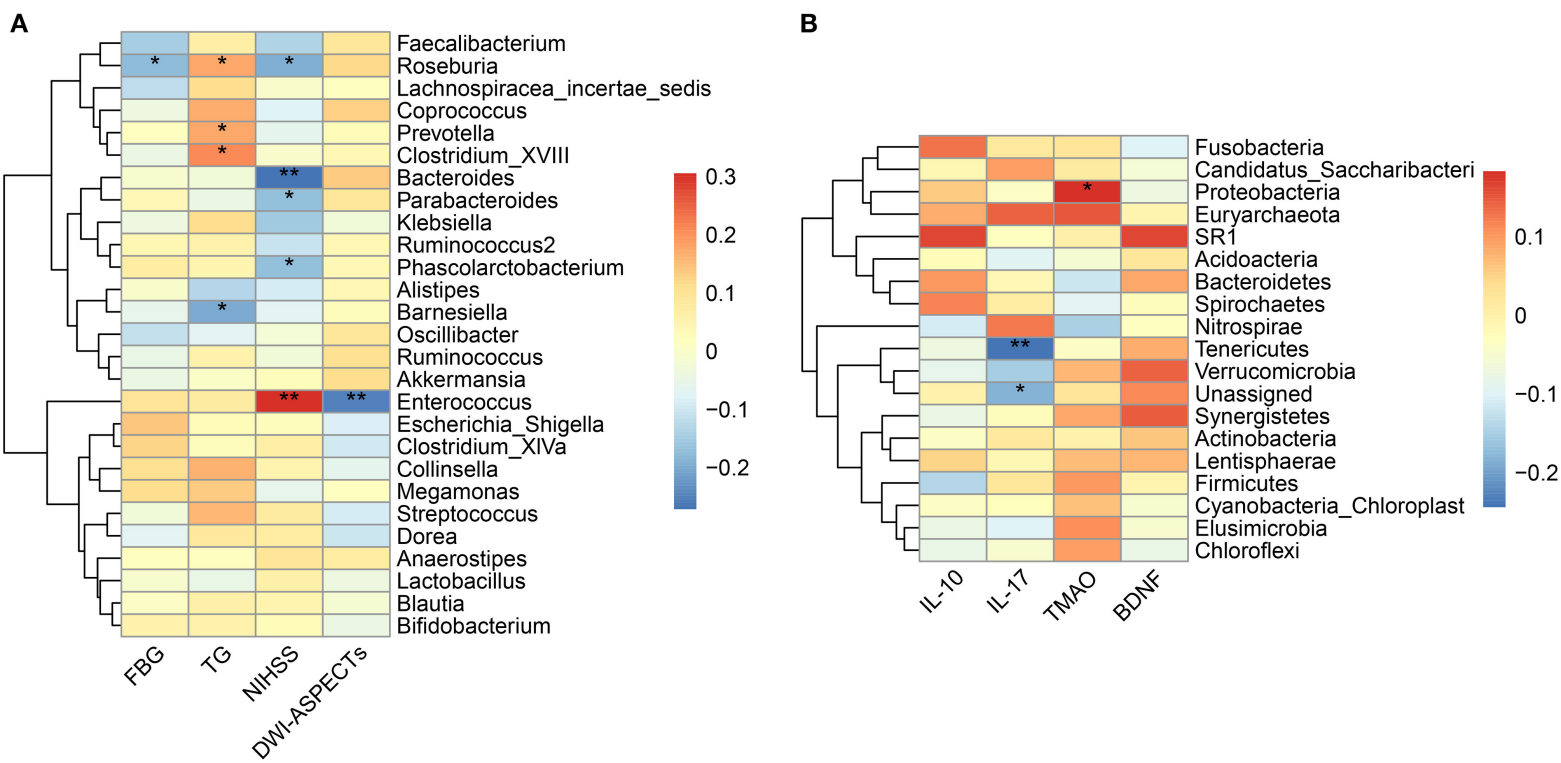
Correlation between the gut microbiota and baseline characteristics. **(A)** Heatmap of Spearman's correlation analysis between bacteria genera and clinical characteristic parameters. **(B)** Heatmap of Spearman's correlation analysis between bacteria phyla and laboratory parameters. *p < 0.05, **p < 0.01. FBG, fasting blood glucose; TG, triglyceride; NIHSS, National Institute of Health Stroke Scale; DWI-ASPECTs, DWI-Alberta Stroke Program Early CT score; IL, interleukin; TMAO, trimethylamine N-Oxide; BDNF, brain-derived neurotropic factor.

As shown in Table 1, The baseline levels of TG, FBG, NIHSS, and DWI-ASPECTs were distributed differently between the two groups. Spearman correlation analyses showed that pathogenic bacterium *Clostridium* was positively correlated with TG (*p* = 0.016), and the SCFAs-producing bacterium *Roseburia* was borderline negatively correlated with FBG (*p* = 0.050). The pathogenic bacterium *Enterococcus* was positively correlated with stroke severity (assessed by NIHSS, *p* < 0.001) and lesion size (estimated by DWI-ASPECTs, *p* = 0.004), while SCFAs-producing bacteria *Bacteroides, Roseburia*, and *Parabacteroides* were negatively correlated with stroke severity (all *p* < 0.05, Figure 4A). All the above-mentioned bacteria, except for *Parabacteroides*, had different abundances between the two groups (all *p* < 0.05).

### TMAO, IL-17, IL-10, and BDNF Levels in Relation to the Gut Microbiota

We did not observe different levels of TMAO, IL-17, IL-10, or BDNF in the mRS 3–6 group when compared to the mRS 0–2 group (Table 1). The results of Spearman correlation analyses showed that the TMAO level was positively correlated with the relative abundances of phylum *Proteobacteria* and family *Enterobacteriaceae* (*p* = 0.050) while negatively correlated with the family *Bacillaceae-1* (Figure 4B, Supplementary Figure S6A). At the genus level, TMAO was positively correlated with *Escherichia/Shigella* (Supplementary Figure S7). The IL-17 level was negatively correlated with phylum *Tenericutes* and positively correlated with genus *Agromyces* (Figure 4B, Supplementary Figure S7). The IL-10 level was positively correlated with family *Bacteroidaceae* and genera *Hungatella* and *Eggerthia* (Supplementary Figures S6B, S7). The BDNF level was positively correlated with genus *Anaerobacterium* (Supplementary Figure S7).

### Biological Function of the Gut Microbiota

**Figure 5:**
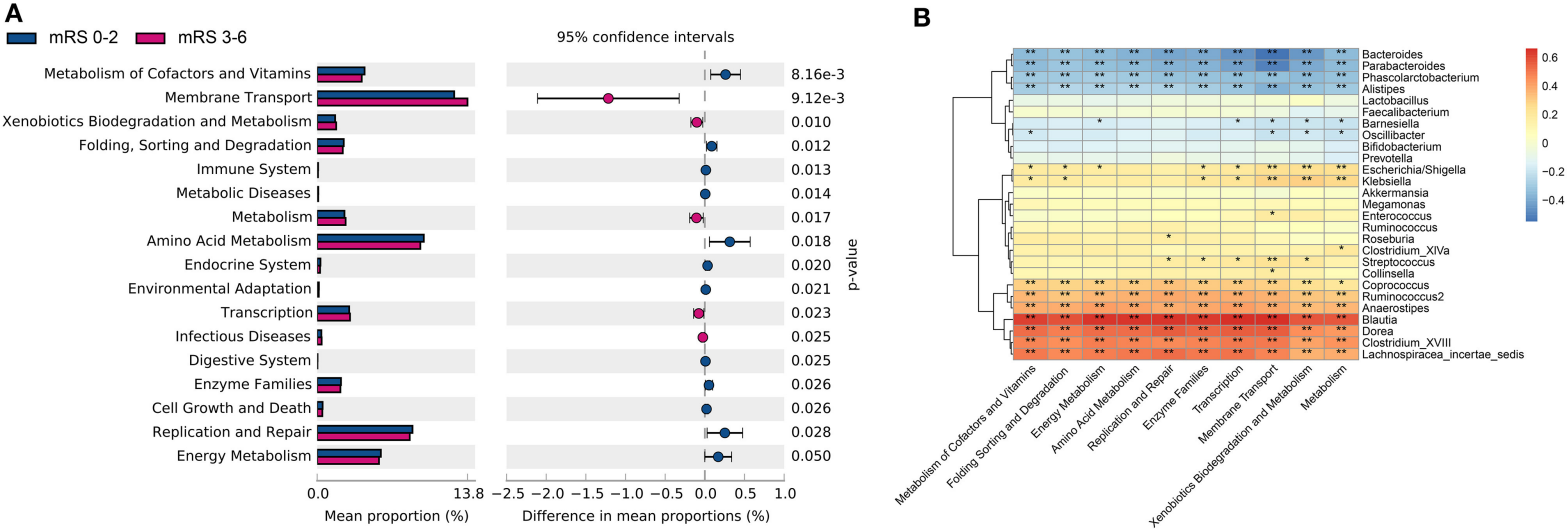
The gut microbiota function differed between the two groups in KEGG pathways and was correlated with bacteria profile. **(A)** Significantly differed microbiota function in KEGG level 2 pathways. **(B)** Heatmap of Spearman's correlation analysis between bacteria genera and the KEGG level 2 pathways. KEGG, kyoto encyclopedia of genes and genomes; mRS, modified Rankin Scale.

The metagenome functional composition identified by PICRUSt showed that in the gut microbiota of patients with stroke with poor outcome, 5 of the 41 Kyoto Encyclopedia of Genes and Genomes (KEGG) pathways (level 2) were upregulated, including membrane transport, transcription, metabolism, xenobiotics biodegradation and metabolism, and infectious diseases (Figure 5A). Whereas, in the gut microbiota of patients with stroke with good outcome, 12 KEGG pathways were upregulated, including amino acid metabolism, metabolism of cofactors and vitamins, enzyme families, replication and repair, etc. At KEGG level 3, 34 pathways relating to metabolism showed significant differences between the two groups, including alanine, aspartate and glutamate metabolism, fructose and mannose metabolism, pyruvate metabolism, among others (Supplementary Figure S8).

Next, the Spearman's correlation analyses between the 10 most differently distributed Kyoto Encyclopedia of Genes and Genomes (KEGG) level 2 pathways and 27 bacteria genera with relative abundance > 1% were performed (Figure 5B). Data showed that the genus *Enterococcus* was positively associated with the membrane transport pathway, while genera *Bacteroides* and *Parabacteroides* were negatively correlated with the membrane transport pathway. Besides, SCFAs-producing bacteria *Ruminococcus* and *Coprococcus* were positively correlated with the amino acid metabolism pathway.

## Discussion

In this study, we found baseline dysbiosis of gut microbiota in patients with acute ischemic stroke with 3-month unfavorable outcome, featured by decreased alpha diversity, increased opportunistic pathogens (e.g., *Enterococcaceae* and *Enterococcus*), and decreased SCFAs-producing bacteria (e.g., *Bacteroidaceae, Ruminococcaceae*, and *Faecalibacterium*) compared to those in the favorable outcome group. Although candidate mediators (i.e., serum TMAO, IL-17, IL-10, and BDNF) were comparable in the acute phase of stroke, there were differences in biological functions such as the membrane transport and amino acid metabolism between the two groups. Our findings showed an association between the baseline alterations of gut microbiota and the functional outcome at 3 months after stroke.

The role played by gut microbiota in stroke pathology has been intensively studied in the recent years. Animal studies suggested that stroke would induce dysfunction of the intestinal barrier, such as disruption of tight junction protein (20), dysregulation of the sympathetic nervous system (21), and impairment of gut motility (22), leading to gut microbiota dysbiosis (15, 23). This may reversely impede neuro-regeneration and enlarge stroke core volume, resulting in poor functional outcome (23). In clinical investigations, a consistently altered profile of gut microbiota has been described in patients with stroke, with an increase of pathogenic bacteria and a decrease of common probiotics (10–14). Moreover, a potential relationship with stroke outcome has been suggested for gut microbiota dysbiosis (11, 12) and this study further emphasized that specific bacteria were in association with unfavorable 3-month stroke outcome.

The decreased alpha diversity in the poor outcome group was observed and similar findings have been reported in patients with other neurological diseases, for instance, Alzheimer's disease and therapy-resistant epilepsy (3). A possible explanation for the decreased alpha diversity observed in this study is that the relative abundance of common core bacteria was decreased due to the “leaky gut” (12, 15, 24), which is characterized by increased intestinal barrier permeability. The stroke mice model found that the supply of SCFAs-producing bacteria increased alpha-diversity and improved outcome (25). Two former studies also found that patients with stroke displayed lower alpha diversity when compared to healthy controls (11, 12), whereas Yin et al. reported elevated alpha diversity, and Li et al. reported no significant difference in alpha diversity between patients with stroke and asymptomatic controls (10, 14). Further studies with a larger sample size might help to verify the prognosis value of alpha diversity in stroke outcome.

In this study, we found the pathogenic bacteria (i.e., *Enterococcus* and *Eggerthella*) were increased in the unfavorable outcome group. Likewise, previous reports indicated higher levels of *Enterococcus* in patients with Parkinson's disease (26) and higher levels of *Eggerthella* in patients with hypertension (6). The pathogenic bacteria have been reported to be positively correlated with early stroke outcome (12), and this study further confirmed a positive correlation between the pathogen *Enterococcus* and the 3-month stroke outcome. *Enterococcus* is a Gram-positive opportunistic pathogen that could cause pneumonia and bacteremia in patients with stroke, worsening functional outcome (20). Mechanically, *Enterococcus* was reported to have interactions with platelets (27) and could modulate neuronal circuits that mediate stress responses (28). It is noteworthy that the pathogenic bacterium *Desulfovibrionaceae*, previously reported to be elevated in patients with stroke and associated with stroke severity (9), showed a decrease in the unfavorable outcome group in this study, which gives rise to the complicated patterns of microbial dysbiosis influenced by the stroke that worth further investigations.

Short-chain fatty acids mainly include acetate, propionate, and butyrate and play an important role in maintaining the gut permeability and blood-brain barriers integrity (15, 22, 29, 30). A lower abundance of SCFAs-producing bacteria was previously demonstrated in brain disorders such as Parkinson's disease and metabolic disorders such as type 2 diabetes (31). In patients with stroke, the decrease of SCFAs-producing bacteria was positively associated with poststroke infection (32) and the reduced SCFAs level was closely linked to an increased risk of 3-month poor functional outcomes (11). In the current study, we found the SCFAs-producing bacteria (e.g., *Bacteroidaceae, Ruminococcaceae*, and *Faecalibacterium*) were decreased in patients with stroke with poor outcome. This finding was in line with former observations that decreased SCFAs-producing bacteria (e.g., *Faecalibacterium, Roseburia*, and *Coprococcus*) predict unfavorable early stroke outcomes (12). Of note, one of the SCFAs, butyrate, which regulates apoptosis and inflammation after stroke (33), may indirectly affect glucose metabolism by interfering with the acetylation of related enzymes (11, 13, 34). Accordingly, we observed an association between the butyrate-producing genus *Roseburia* and FBG. In total, our findings suggest that the SCFAs-producing bacteria are important for stroke outcome. Further studies focusing on the neuroprotective value of SCFAs-producing bacteria might provide novel therapeutic targets for patients with stroke (23, 25).

Disrupted microbiota composition accompanied by an elevated level of proinflammatory IL-17 and a decreased level of neuroprotective IL-10 were observed in stroke mice (22), and an elevated TMAO level was reported in patients with acute ischemic stroke (35). Nevertheless, this study showed that stroke outcome could not be differentiated by the serum levels of TMAO, IL-17, or IL-10. This result should be interpreted with caution as we collected the blood samples mostly within 72 h after the stroke onset, while the poststroke inflammatory response was reported to peek at 3–5 days (22), and the TMAO might change dynamically after stroke, with an elevation within 24 h and a significant decrease thereafter (36). There is, however, an association between TMAO and the pathogenic bacterium *Enterobacteriaceae* in the light of previous observations that pathogenic bacteria would worsen disease outcome by regulating TMAO generation (18).

In patients with stroke with poor outcome, the functional alterations of elevated membrane transport suggested intestinal mucosal microbiota disruption (37), and the upregulated enzymatic pathway of xenobiotic metabolism/aromatic biodegradation has been shown to predict poor stroke outcome in the mice model as well (23). On the other hand, the pathway associated with amino acid metabolism, which could be of neuroprotective value (23), was elevated in the favorable outcome group. Furthermore, we found a positive correlation between the genus *Enterococcus* and the membrane transport pathway, as well as between SCFAs-producing bacteria (i.e., *Ruminococcus* and *Coprococcus*) and the amino acid metabolism pathway. Further studies are needed to test the possibility of specific bacteria affecting stroke outcome through these pathways.

This study has several limitations. First, it was a single-center study with a small sample size and is limited to the Chinese Han population. Second, dietary factors were not taken into consideration when comparing the composition of gut microbiota between the two groups. Previous studies have shown that long-term diet affects the fecal microbial community, especially protein and animal fat (*Bacteroides*) and carbohydrates (*Prevotella*) (38). However, these hospitalized participants were local residents with similar levels of dietary structure. Third, the participants differed in age, the NIHSS score, and a history of hypertension, which may lead to sampling bias (2, 7). Although we performed PSM analysis to obtain matched pairs, and the results fitted well, further information would be needed from validation cohorts including new patients. Finally, it is widely believed that SCFAs are associated with stroke outcome (23, 24, 30), which we however have not included in the testing of fecal or blood samples.

In conclusion, this study suggested the gut microbiota in the acute phase of stroke had an association with the functional outcome at 3 months. Patients with stroke with 3-month unfavorable outcome are characterized by an increase of pathogenic bacteria and a decrease of SCFAs-producing bacteria, as well as the upregulated membrane transport and xenobiotics biodegradation pathways. Additional multicenter studies with larger sample sizes are needed to validate our findings, as well as to verify if the conclusions are confined to a specific region or are general.

## Data Availability Statement

The datasets presented in this study can be found in online repositories. The name of the repository and accession number can be found at: Genome Sequence Archive (GSA), https://ngdc.cncb.ac.cn/gsa/, CRA005254.

## Ethics Statement

The studies involving human participants were reviewed and approved by the Ethical Review Board of Nanjing First Hospital (Nanjing, China). The patients/participants provided their written informed consent to participate in this study.

## Author Contributions

HS, MG, and XC conceived the study. HS and MG analyzed the data. HS, MG, and ZL interpreted the data. HS wrote the manuscript. XC and JZ revised the manuscript.

## Funding

This study was supported by the National Nature Science Foundation of China (81701064), the Medical Science and Technology Program of Nanjing (JQX20007) and National Science and Technology Innovation 2030 – Major program of “Brain Science and Brain-Inspired Intelligence Research” (2021ZD0201807).

## Conflict of Interest

The authors declare that the research was conducted in the absence of any commercial or financial relationships that could be construed as a potential conflict of interest.

## Publisher's Note

All claims expressed in this article are solely those of the authors and do not necessarily represent those of their affiliated organizations, or those of the publisher, the editors and the reviewers. Any product that may be evaluated in this article, or claim that may be made by its manufacturer, is not guaranteed or endorsed by the publisher.
