# Supplementary material for: Gut Microbiota Dysbiosis in Acute Ischemic Stroke Associated With 3-Month Unfavorable Outcome
Source: Front Neurol. 2022 Jan 28;12:799222. doi: 10.3389/fneur.2021.799222 (PMC8831883; doi:10.3389/fneur.2021.799222)
Supplement: Supplementary file 1 [file Table_1.docx]

**­­Supplementary materials**

**Supplementary figure S1. Flow chart.**

**
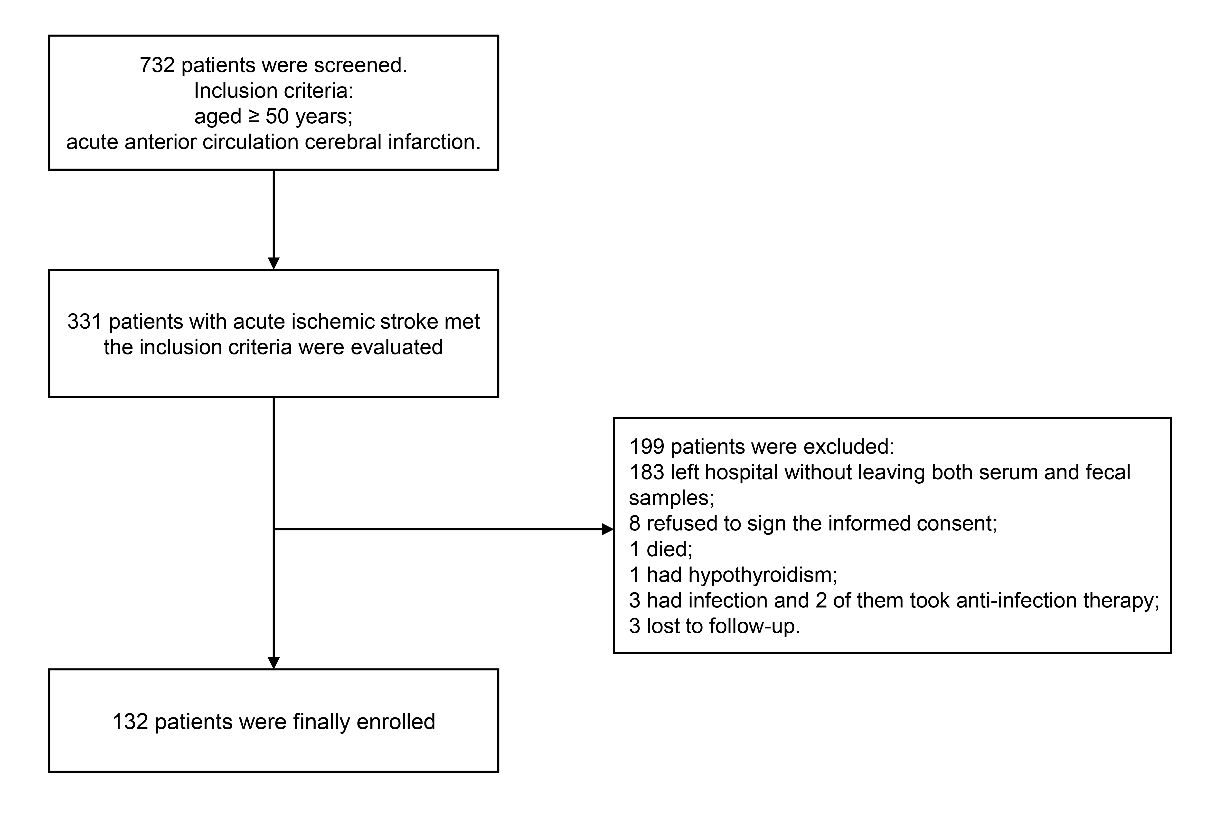
**


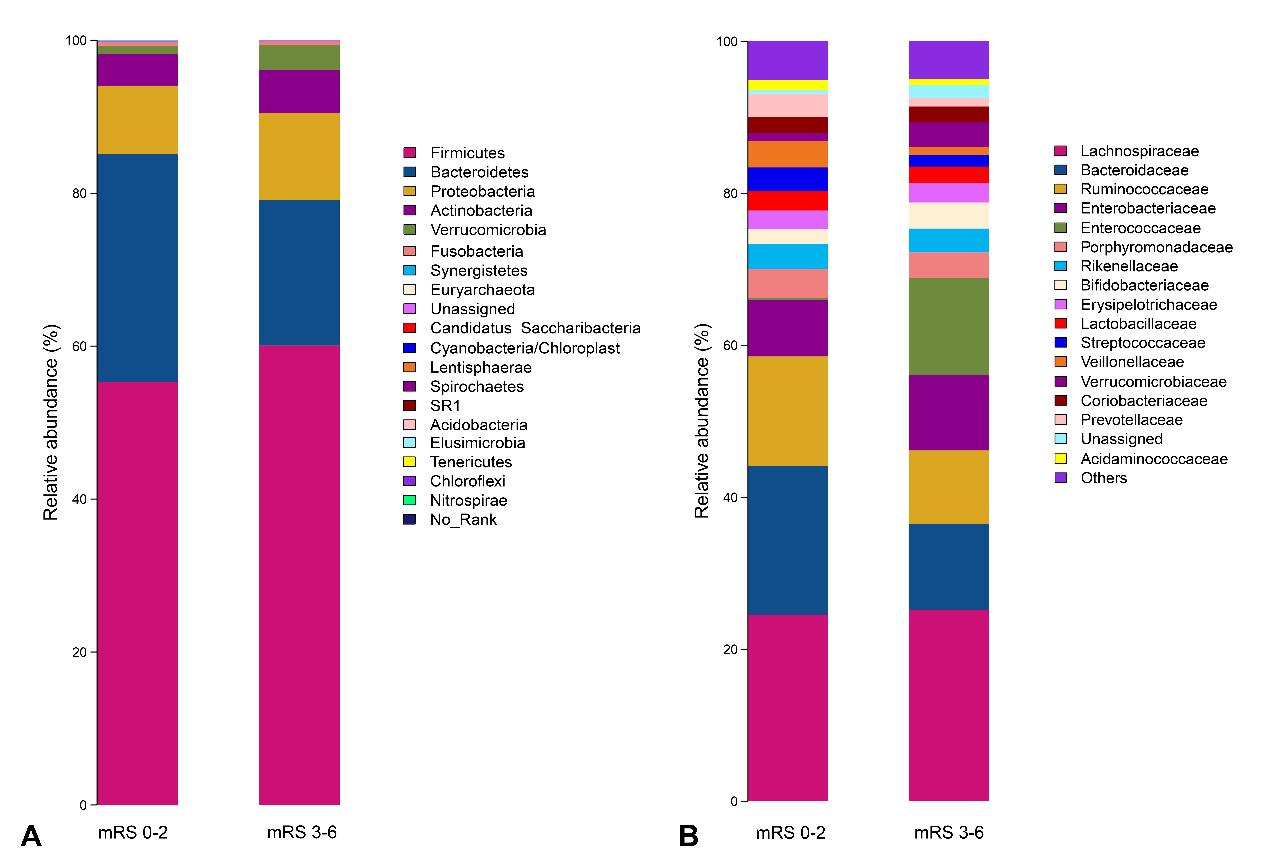
**Supplementary figure S2.** The average relative abundance of dominant microbes in the mRS 0-2 and mRS 3-6 groups at the phylum (A) and family (B) levels, and each color represents a taxon.

Abbreviation: mRS = modified Rankin Scale.


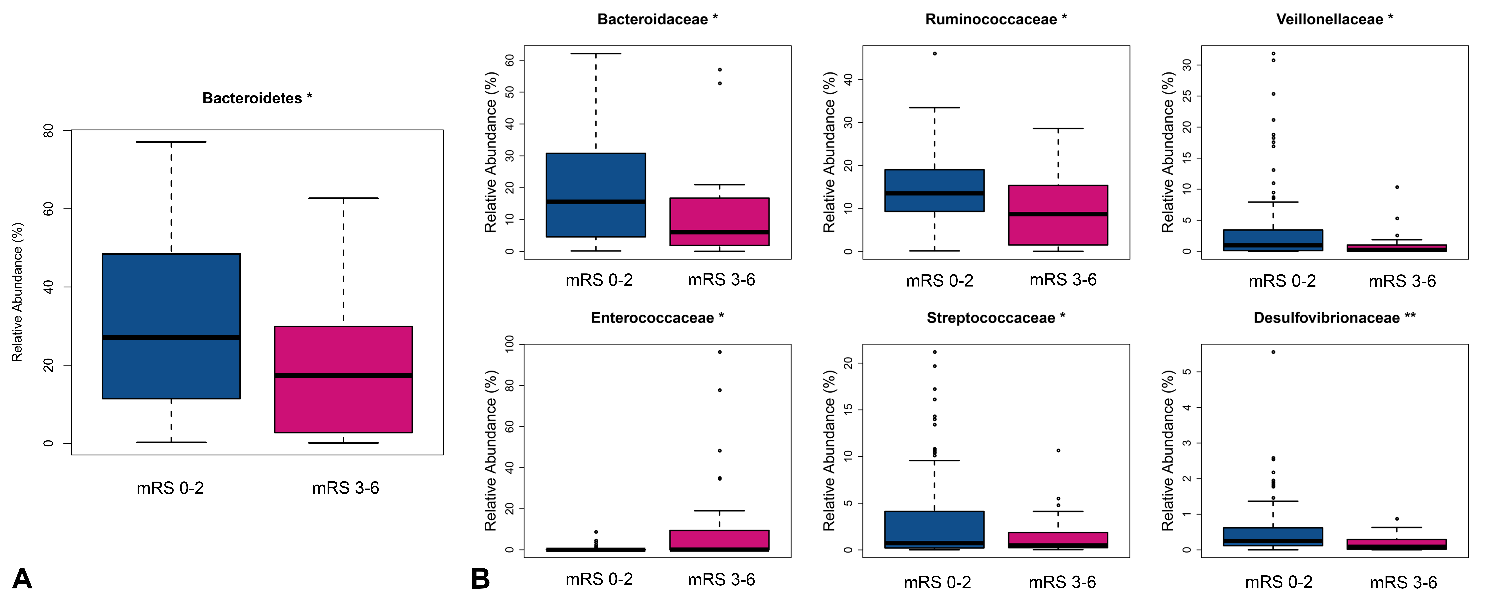
­­**Supplementary figure S3.** Taxa with significantly different relative abundances at phylum (A) and family (B) levels.

Abbreviation: mRS = modified Rankin Scale.

**Supplementary figure S4.** Bacteria genera with significantly different relative abundances at the genus level after PSM.


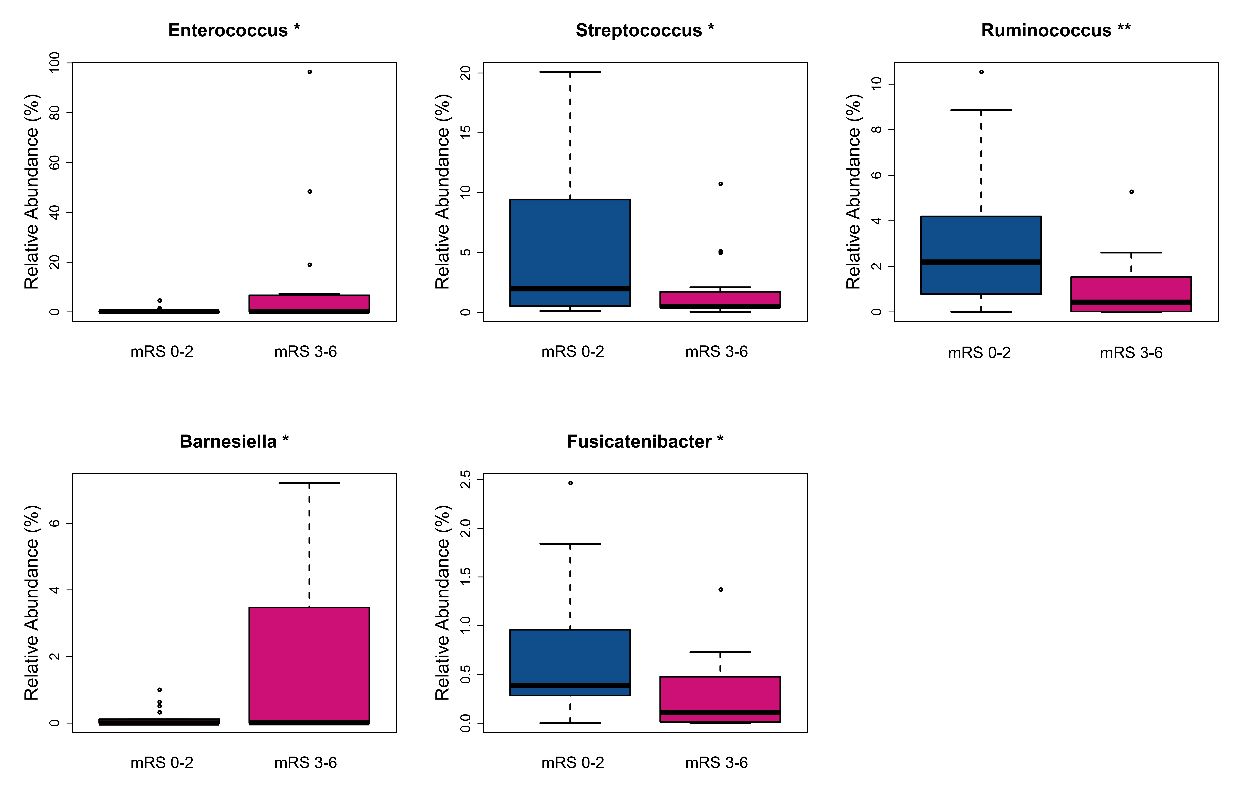


Abbreviations: PSM = propensity score-matched analysis; mRS = modified Rankin Scale.

**Supplementary figure S5.** Significantly discriminative taxa between good outcome and poor outcome patients determined using LEfSe after PSM.


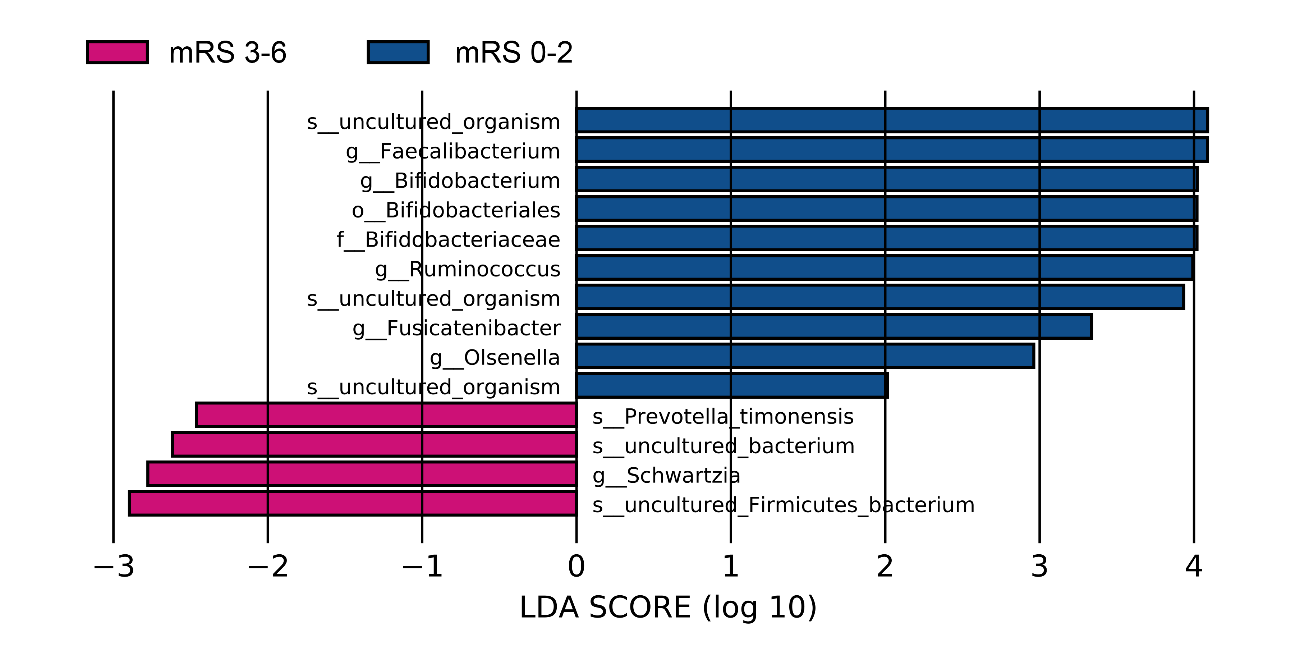


Abbreviations: LEfSe = linear discriminate analysis size effect; PSM = propensity score-matched analysis; LDA = linear discriminate analysis; mRS = modified Rankin Scale.

**Supplementary figure S6.** Heatmap of spearman correlation analysis between microbiota and laboratory indices at the family level before (A) and after (B) PSM.


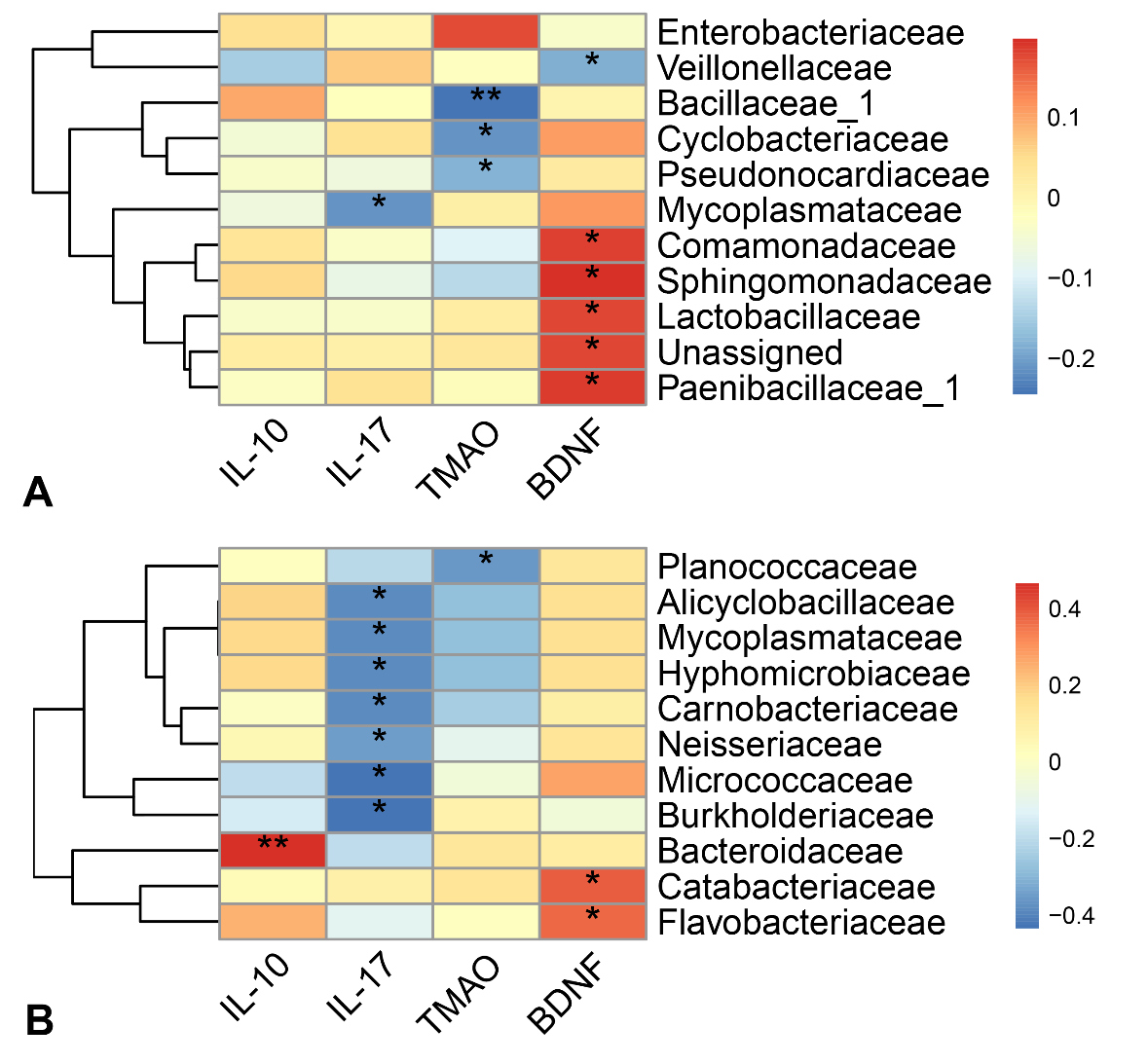


Abbreviations: PSM = propensity score-matched analysis; IL = interleukin; TMAO = trimethylamine N-Oxide; BDNF = brain derived neurotropic factor.

**Supplementary figure S7.** Heatmap of spearman correlation analysis between microbiota and laboratory indices at the genus level (before PSM).


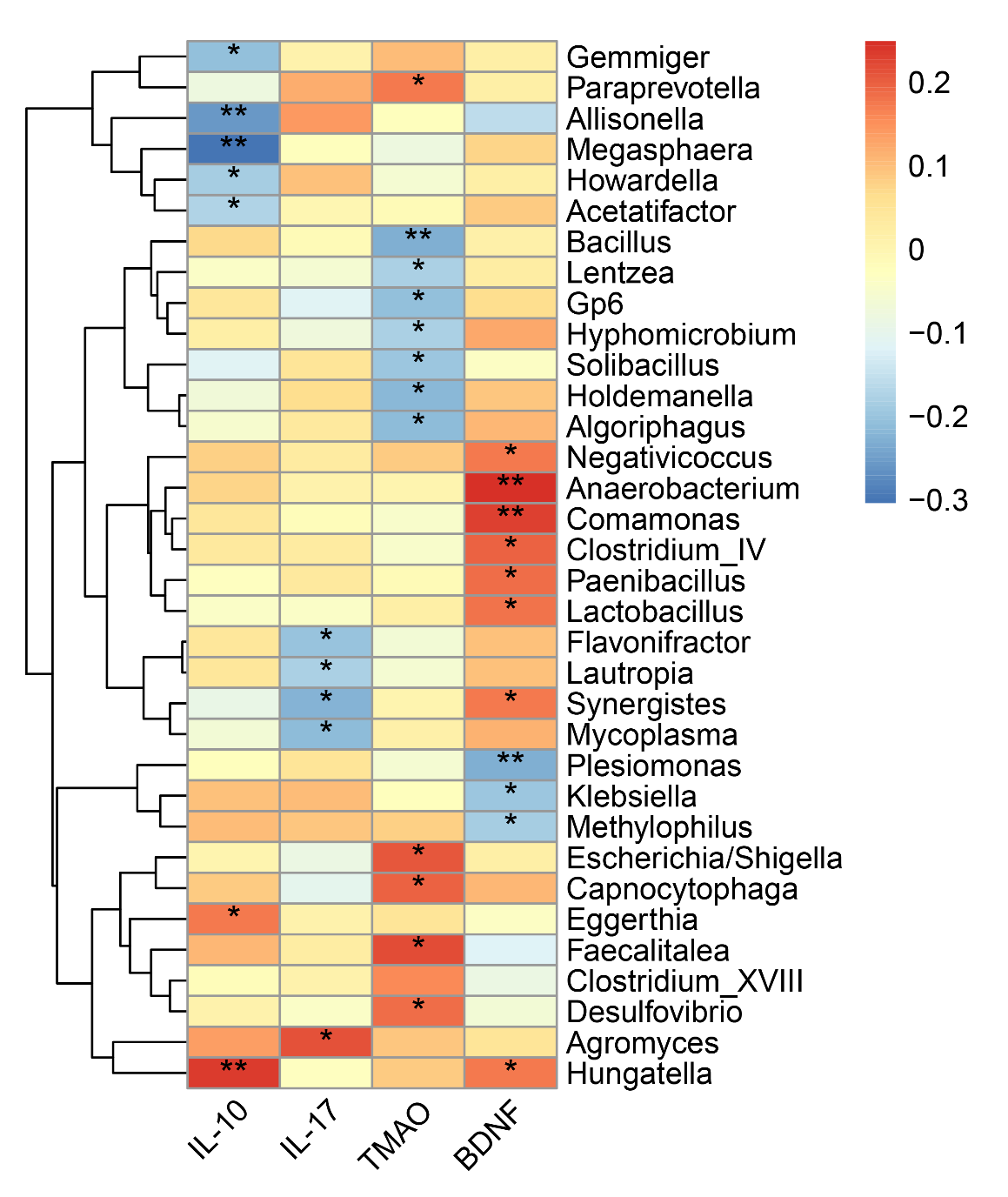


Abbreviations: PSM = propensity score-matched analysis; IL = interleukin; TMAO = trimethylamine N-Oxide; BDNF = brain derived neurotropic factor.

**Supplementary figure S8.** Significantly differed microbiota function relating to metabolism between the mRS 0-2 and mRS 3-6 groups in KEGG level 3 pathways.

**
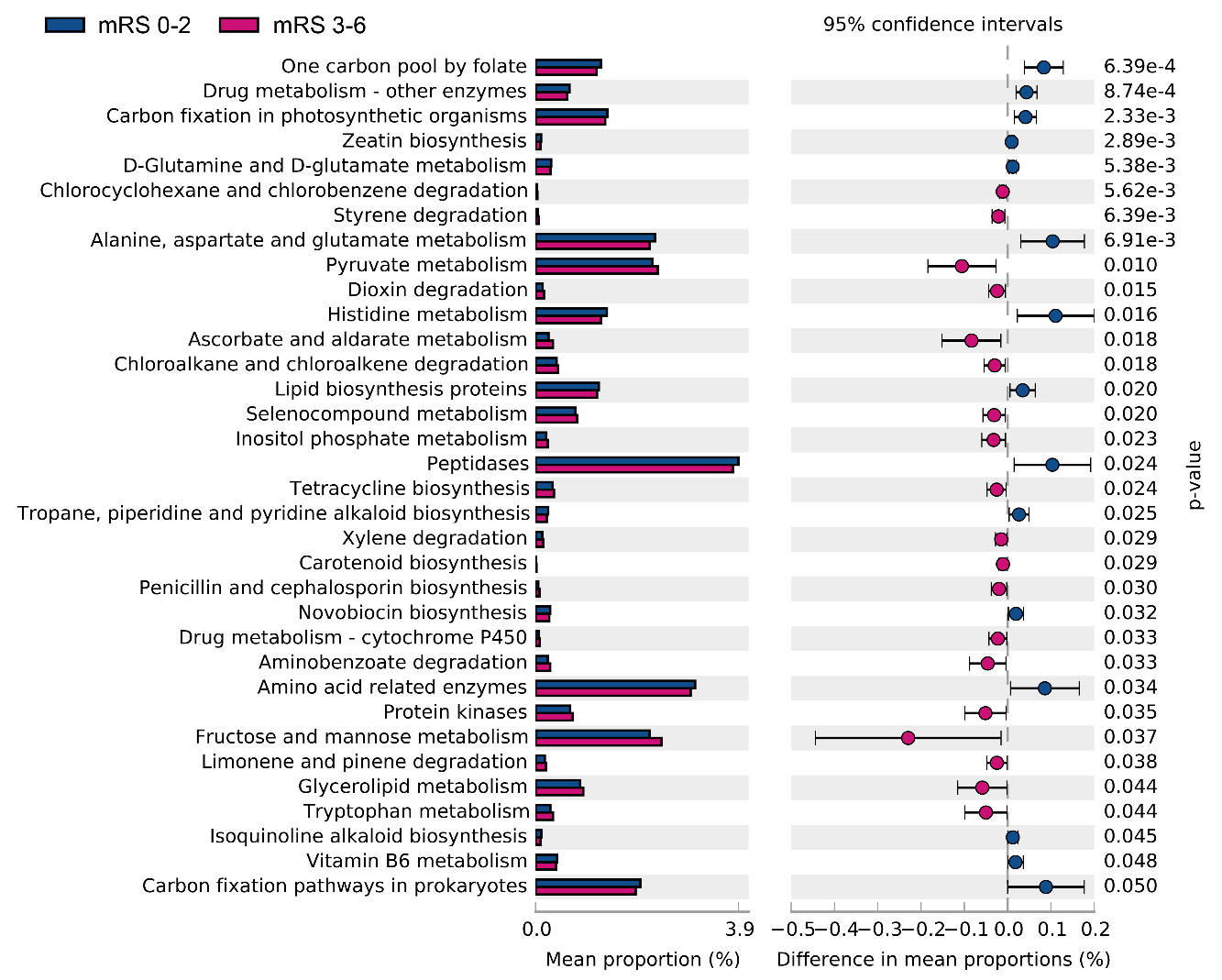
**

Abbreviations: mRS = modified Rankin Scale; KEGG = Kyoto Encyclopedia of Genes and genomes.
